# International consensus on the management of metastatic gastric cancer: step by step in the foggy landscape: Bertinoro Workshop, November 2022

**DOI:** 10.1007/s10120-024-01479-5

**Published:** 2024-04-18

**Authors:** Paolo Morgagni, Maria Bencivenga, Fatima Carneiro, Stefano Cascinu, Sarah Derks, Maria Di Bartolomeo, Claire Donohoe, Clarisse Eveno, Suzanne Gisbertz, Peter Grimminger, Ines Gockel, Heike Grabsch, Paulo Kassab, Rupert Langer, Sara Lonardi, Marco Maltoni, Sheraz Markar, Markus Moehler, Daniele Marrelli, Maria Antonietta Mazzei, Davide Melisi, Carlo Milandri, Paul Stefan Moenig, Bianca Mostert, Gianni Mura, Wojciech Polkowski, John Reynolds, Luca Saragoni, Mark I. Van Berge Henegouwen, Richard Van Hillegersberg, Michael Vieth, Giuseppe Verlato, Lorena Torroni, Bas Wijnhoven, Guido Alberto Massimo Tiberio, Han-Kwang Yang, Franco Roviello, Giovanni de Manzoni, Bertinoro Workshop Working Group

**Affiliations:** 1 Department of General Surgery, Morgagni-Pierantoni Hospital, Forlì, Italy; 2 General and Upper GI Surgery, Department of Surgery, University Hospital Verona, University of Verona, Verona, Italy; 3 Department of Pathology, Centro Hospitalar de São João, Institute of Molecular Pathology and Immunology of the University of Porto (Ipatimup), Porto, Portugal; 4 Department of Medical Oncology, Comprehensive Cancer Center, Università Vita-Salute, IRCCS Ospedale San Raffaele, Milan, Italy; 5 Department of Medical Oncology, Amsterdam UMC, Vrije Universiteit Amsterdam, Amsterdam, The Netherlands; 6 Department of Medical Oncology, Fondazione IRCCS Istituto Nazionale dei Tumori, Milan, Italy; 7 Medicinal Chemistry, Trinity Translational Medicine Institute, Trinity Centre for Health Sciences, Trinity College Dublin, The University of Dublin, St. James’s Hospital, Dublin 8, Ireland; 8 Department of Digestive and Oncologic Surgery, Claude Huriez University Hospital, Centre Hospitalier Universitaire (CHU) Lille, Université de Lille, Lille, France; 9 Department of Surgery, Cancer Center Amsterdam, Amsterdam UMC, University of Amsterdam, Amsterdam, The Netherlands; 10 Department of General, Visceral and Transplant Surgery, University Medical Center, University of Mainz, Mainz, Germany; 11 Department of Visceral, Transplant, Thoracic and Vascular Surgery, University Hospital of Leipzig, Leipzig, Germany; 12 Department of Pathology, GROW School for Oncology and Developmental Biology, Maastricht University Medical Center, Maastricht, The Netherlands; 13 Pathology and Data Analytics, Leeds Institute of Medical Research at St James’s, University of Leeds, Leeds, United Kingdom; 14 Gastric Surgery Division, BP Gastric Surgery Department, Santa Casa Medical School, São Paulo, Brazil; 15 Institute of Pathology and Microbiology, Johannes Kepler University Linz, Altenberger Strasse 69, 4040 Linz, Austria; 16 Istituto Oncologico Veneto IOV-IRCCS, Padua, Italy; 17 Unit of Palliative Care, Istituto Scientifico Romagnolo per lo Studio e la Cura dei Tumori (IRST) IRCCS, Meldola, Forlì-Cesena Italy; 18 Surgical Interventional Trials Unit, University of Oxford, Oxford, UK; 19 Department of Medicine, Johannes-Gutenberg University Clinic, Mainz, Germany; 20 Unit of General Surgery and Surgical Oncology, Department of Medicine Surgery and Neurosciences, University of Siena, 53100 Siena, Italy; 21 Unit of Diagnostic Imaging, Department of Medical, Surgical and Neuro Sciences and of Radiological Sciences, Azienda Ospedaliero-Universitaria Senese, University of Siena, 53100 Siena, Italy; 22 Medical Oncology at the Department of Medicine, University of Verona, Verona, Italy; 23 Department of Oncology, San Donato Hospital, 52100 Arezzo, Italy; 24 Surgery Department, Geneva University Hospitals, Geneva, Switzerland; 25 Department of Medical Oncology, Erasmus MC Cancer Institute, Dr. Molewaterplein 40, 3015 GD Rotterdam, The Netherlands; 26 Department of Surgery, San Donato Hospital, Arezzo, Italy; 27 Department of Surgical Oncology, Medical University of Lublin, Radziwiłłowska 13 St, 20-080 Lublin, Poland; 28 Trinity College, Dublin, Ireland; 29 Pathology Unit, Santa Maria delle Croci Ravenna Hospital, Ravenna, Italy; 30 University Medical Center Utrecht, Heidelberglaan 100, 3584 CX Utrecht, The Netherlands; 31 Institute of Pathology, Klinikum Bayreuth, Bayreuth, Germany; 32 Department of Diagnostics and Public Health, Section of Epidemiology and Medical Statistics, University of Verona, Verona, Italy; 33 Department of Surgery, Erasmus MC-University Medical Centre Rotterdam, Rotterdam, Netherlands; 34 Surgical Clinic, Department of Clinical and Experimental Sciences, University of Brescia, Brescia, Italy; 35 Surgical Department, SNUH National Cancer Center, Seoul, Korea

**Keywords:** Stage IV, Oligometastatic gastric cancer, Consensus, Staging, Multimodal treatment

## Abstract

**Background:**

Many gastric cancer patients in Western countries are diagnosed as metastatic with a median overall survival of less than twelve months using standard chemotherapy. Innovative treatments, like targeted therapy or immunotherapy, have recently proved to ameliorate prognosis, but a general agreement on managing oligometastatic disease has yet to be achieved. An international multi-disciplinary workshop was held in Bertinoro, Italy, in November 2022 to verify whether achieving a consensus on at least some topics was possible.

**Methods:**

A two-round Delphi process was carried out, where participants were asked to answer 32 multiple-choice questions about CT, laparoscopic staging and biomarkers, systemic treatment for different localization, role and indication of palliative care. Consensus was established with at least a 67% agreement.

**Results:**

The assembly agreed to define oligometastases as a “dynamic” disease which either regresses or remains stable in response to systemic treatment. In addition, the definition of oligometastases was restricted to the following sites: para-aortic nodal stations, liver, lung, and peritoneum, excluding bones. In detail, the following conditions should be considered as oligometastases: involvement of para-aortic stations, in particular 16a2 or 16b1; up to three technically resectable liver metastases; three unilateral or two bilateral lung metastases; peritoneal carcinomatosis with PCI ≤ 6. No consensus was achieved on how to classify positive cytology, which was considered as oligometastatic by 55% of participants only if converted to negative after chemotherapy.

**Conclusion:**

As assessed at the time of diagnosis, surgical treatment of oligometastases should aim at R0 curativity on the entire disease volume, including both the primary tumor and its metastases. Conversion surgery was defined as surgery on the residual volume of disease, which was initially not resectable for technical and/or oncological reasons but nevertheless responded to first-line treatment.

**Supplementary Information:**

The online version contains supplementary material available at 10.1007/s10120-024-01479-5.

## Introduction

Gastric cancer is still one of the leading causes of cancer-related deaths worldwide, with over one million of new cases in 2020 and an estimated 769,000 deaths, ranking fifth for incidence and fourth for mortality globally [1].

Due to the lack of screening programs in the West, 35–55% of patients present with metastatic disease at diagnosis [2] with a median overall survival of 9–11 months when treated with standard chemotherapy [3]. However, in recent years there has been a growing effectiveness of systemic therapy for metastatic patients both for the introduction of new chemotherapy schemes, target therapy, and immunotherapy, and for the optimization of the patient's general conditions.

At the same time, the evidence has grown that a subgroup of metastatic patients is in a transitional state between localized and widespread disease, this can be defined as oligometastatic gastric cancer (OGC) [4].

Oligometastatic GC is characterized by limited tumor burden. An aggressive multimodal integrated approach for such cases, including both systemic therapy and local ablative treatment after response to systemic therapy, demonstrates a non-negligible survival (about 31 months) that is significantly higher than that of poly metastatic GC undergoing systemic chemotherapy alone [3, 5].

The biological mechanisms of transition between oligo and poly metastatic disease are currently not well known. However, it is conceivable that in some cases resection of oligo metastases may prevent further dissemination of disease allowing survival benefit. The only way to select these patients, currently, is to observe their response to chemotherapy over time. Therefore, the definition of oligo metastatic disease should consider both the burden of disease at diagnosis and the response to chemotherapy [3, 6–8].

It is important to note that an improved prognosis was observed even in extremely selected patients with poly metastases treated with surgery after response to systemic therapy.

In this context, a major issue is to achieve a clear and shared definition of oligo and poly metastatic gastric cancer to be used in clinical practice as well as a definite treatment path. Recently, the attention to this specific issue has grown a lot and some projects have been designed to deal with it, but there is still no comprehensive and globally shared evidence on these topics [9–11]. It should be underlined that evidence available from the current literature is limited, as published randomized trials are still lacking.

The present project aimed at discussing the current status of diagnosis and treatment of synchronous metastatic gastric cancer (including Siewert 3, but excluding the other EGJ and esophageal tumors) by a multidisciplinary international team of specialists and trying to reach agreement on the definition and clinical pathway to be followed or identify areas for further research.

## Methods

The methodology of this project was similar to that of other multicentric consensus reports [12, 13]. First, possible guidelines for metastatic gastric cancer were proposed by a central team of members, following a literature search. Second, a formal multi-disciplinary process was designed using a Delphi method, which involved two anonymous rounds.

A restricted working group (RWG) of the Italian Research Group for Gastric Cancer (GIRCG), composed of two oncologists, five surgeons, one radiologist, one pathologist and one biostatistician, established the project's aim and defined the topics for debate. The RWG generated statements based on the controversial results from the current medical literature identifying areas of uncertainty about defining and managing stage IV gastric cancers. Based on the controversial literature, a multiple-choice questionnaire was developed. It consisted of a total of 32 questions (see Table 1), divided into three macro-areas:

A. **How to stage (questions 1–8):** relating to the type of imaging used for diagnosis of gastric cancer (and metastatic disease), the role of laparoscopic staging, and biomarkers needed for treatment.
B. **How to treat (questions 9–29):** relating to definition and local and systemic treatment for different types of metastases (lung, bone, lymph nodes, peritoneal…).
C. **How to care (questions 30–32):** relating to role and indication for palliative care.

**Table 1:**
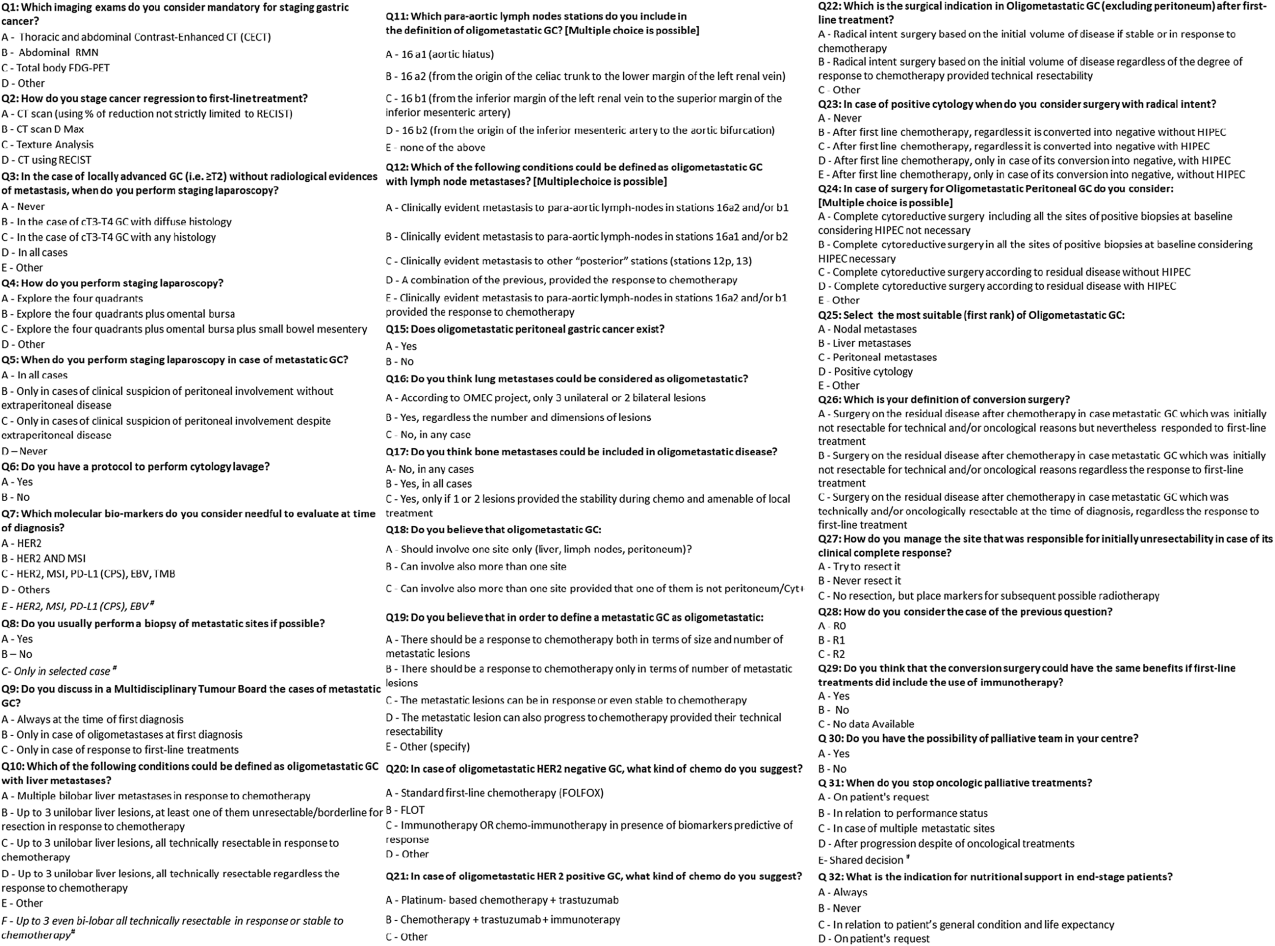
Summary questionnaire

^#^New answer after second round discussion

The RGW selected the expanded working group (EWG) of Worldwide experts in gastric cancer surgery and interpreted the results. Members of the EWG, who agreed to participate and got involved in the rating process, are listed in Supplementary Table 1.

In the first round, the questionnaire was sent out by e-mail to 96 experts. Answers were anonymously collected from June the 6th to the 1st of September 2022 through an online survey system. The first round was completed by 52 members. Thereafter, the EGW was invited to participate in a dedicated workshop held in Bertinoro, Italy, on the 10th of November 2022 for the second-round, which was attended by 78 experts.

Each expert was asked to comment and suggest modifications to the draft statements through a Delphi method implementation. During the workshop, the existing evidence in the literature on each previously identified topic was presented by two experts and the specific topics and the answers given to the first-round questionnaire were discussed in the plenary. Finally, the questionnaire was administered for the second round and a draft Statement in response to each issue was recorded.

The answers of the experts were anonymously collected and reported below as percentages for each multiple-choice question. The RGW decided a priori the minimum cut-off level for Consensus that was two-thirds (≥ 67%) of agreement of effective answers [14–16]. The agreement was further categorized as satisfactory (67–69%), good (70–79%), excellent (80–89%) and exceptional (≥ 90%) [17].

The classic GRADE approach could not be used, as evidence from the literature was sparse and published randomized clinical trials were lacking [18–20]. Most studies on this topic are observational retrospective, but one non-randomized prospective phase 2 study (AIO-FLOT3) [5]. Indeed, a randomized phase III trial (AIO-FLOT5) is ongoing, aimed at comparing chemotherapy alone vs. chemotherapy followed by surgical resection in patients with limited-metastatic adenocarcinoma of the stomach or esophagogastric junction [21], but the results have not been published yet.

## Results

### (A) How to stage

1. Which imaging exams do you consider mandatory for staging gastric cancer?
  - Thoracic and abdominal Contrast-Enhanced CT (CECT) **(100%)**
  - b. Abdominal MRI **(0%)**
  - Total body FDG-PET **(0%)**
  - Other **(0%)**

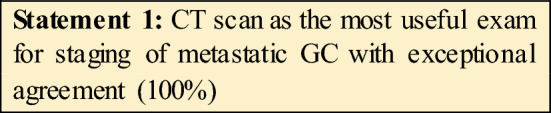



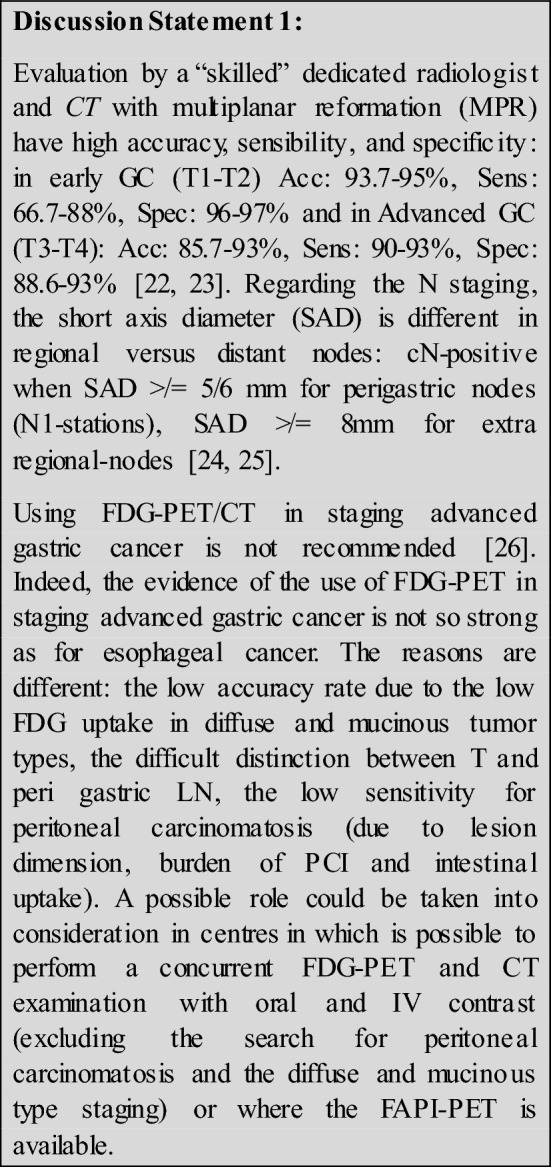

2. How do you stage cancer regression to first-line treatment?
  - CT scan (using % of reduction not strictly limited to RECIST) **(41%)**
  - b. CT scan D Max **(0%)**
  - Texture Analysis **(0%)**
  - CT using RECIST **(59%)**

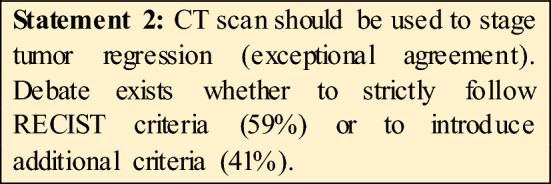



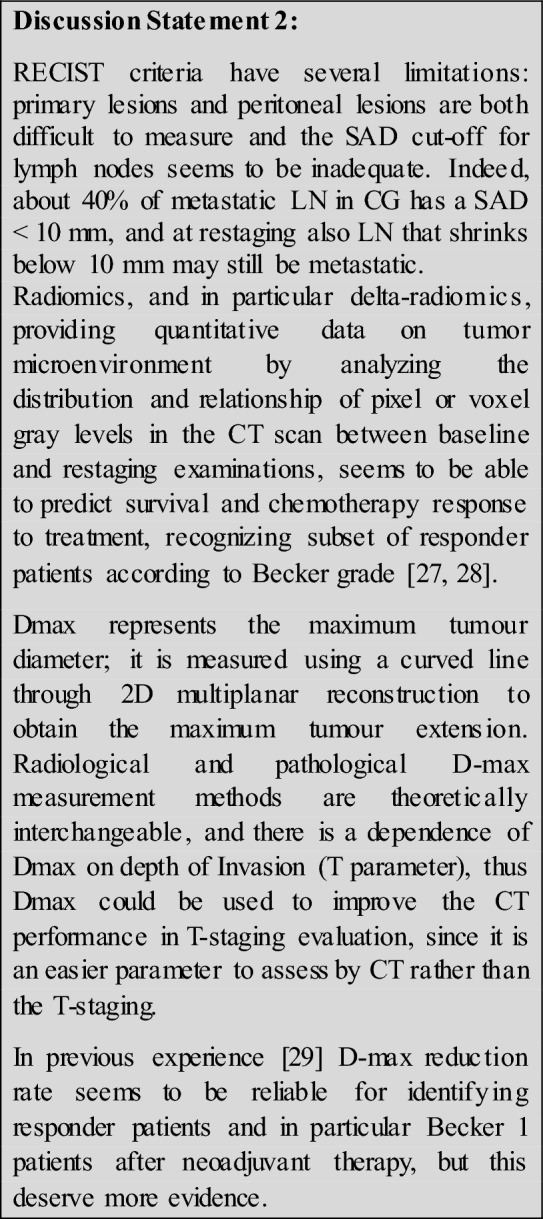

3. In the case of locally advanced GC (i.e., ≥ T2) without radiological evidence of metastasis, when do you perform staging laparoscopy?
  - Never **(3%)**
  - In the case of cT3-T4 GC with diffuse histology **(3%)**
  - In the case of cT3-T4 GC with any histology **(35%)**
  - In all cases **(56%)**
  - Other **(3%)**
4. How do you perform staging laparoscopy?
  - Explore the four quadrants **(14%).**
  - Explore the four quadrants plus omental bursa **(8%).**
  - Explore the four quadrants plus omental bursa plus small bowel mesentery **(59%).**
  - Other—please specify **(19%).**
5. When do you perform staging laparoscopy in case of metastatic GC?
  - In all cases **(6%).**
  - b. Only in cases of clinical suspicion of peritoneal involvement without the extraperitoneal disease **(83%).**
  - Only in cases of clinical suspicion of peritoneal involvement despite extraperitoneal disease **(3%).**
  - Never **(8%).**
6. Do you have a protocol to perform cytology lavage?
  - Yes (please specify) **(43%)**
  - No (57%)

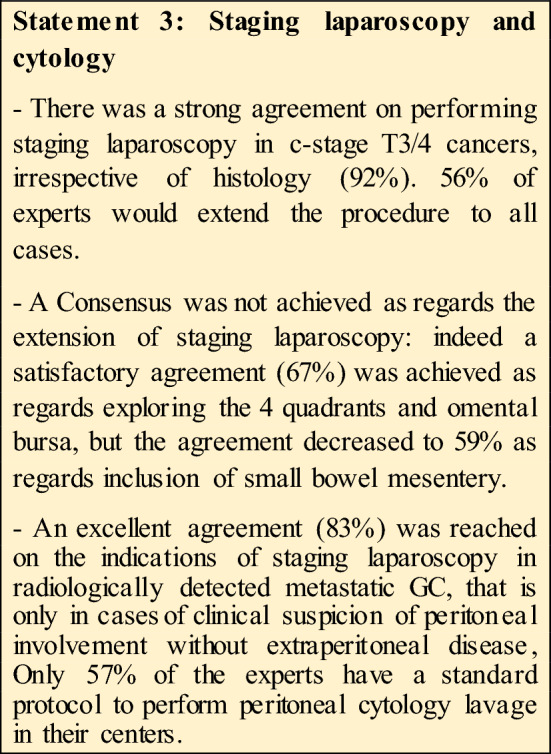



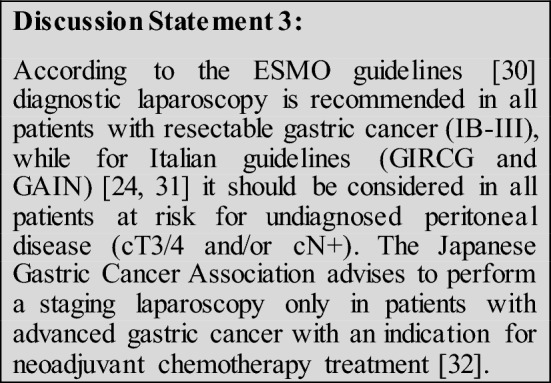

7. Which molecular biomarkers do you consider necessary to test at time of diagnosis of metastatic gastric cancer ?
  - HER2 **(2%)**
  - HER2 and MSI **(7%)**
  - HER2, MSI, PD-L1 (CPS), EBV, TMB **(16%)**
  - Others, please specify **(2%)**
  - HER2, MSI, PD-L1 (CPS), EBV (**new option added**) **(73%)**

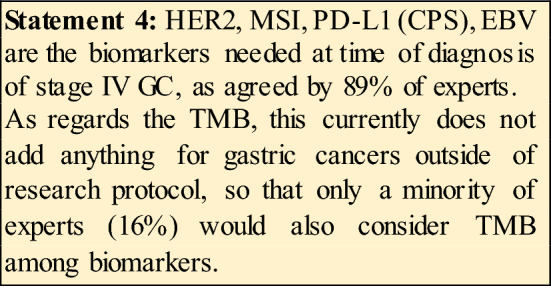



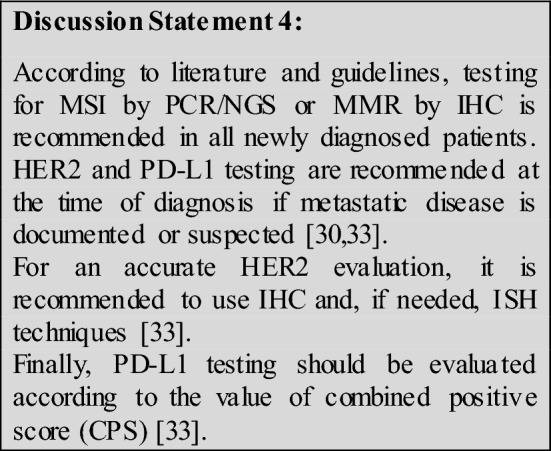

8. Do you usually perform a biopsy of metastatic sites if possible?
  - Yes (31%).
  - b. No **(21%).**
  - Only in selected cases (**new option added**) **(48%).**

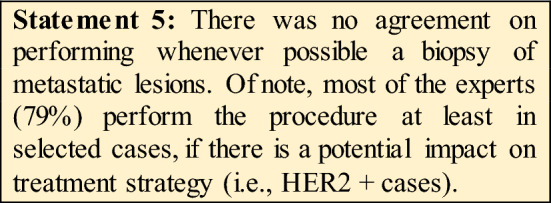



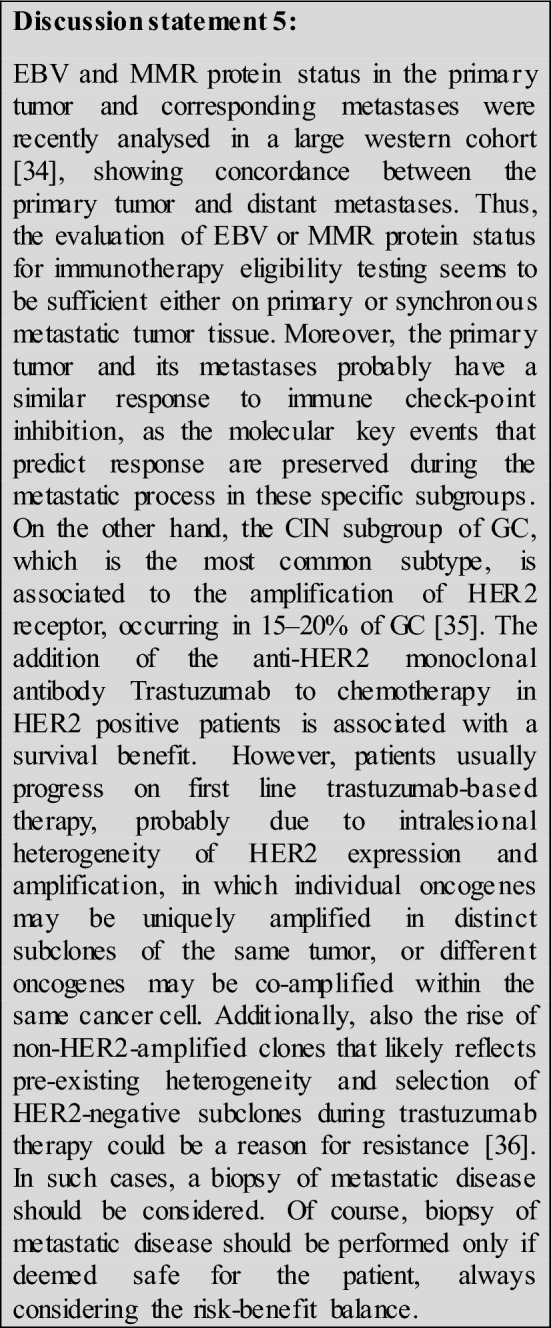


### (B) How to treat

9. Do you discuss in a Multidisciplinary Tumour Board the cases of metastatic GC?
  - Always at the time of first diagnosis **(85%)**
  - Only in case of oligometastases at first diagnosis **(4%)**
  - Only in case of response to first-line treatments **(11%)**

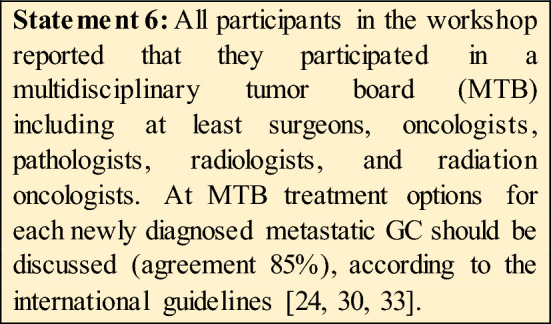

10. Which of the following conditions could be defined as oligometastatic GC with liver metastases?
  - Multiple bilobar liver metastases in response to chemotherapy **(2%)**
  - Up to 3 unilobar liver lesions, at least one of them unresectable/borderline for resection in response to chemotherapy **(5%)**
  - Up to 3 unilobar liver lesions, all technically resectable in response to chemotherapy **(50%)**
  - Up to 3 unilobar liver lesions, all technically resectable regardless of the response to chemotherapy **(7%)**
  - Other **(5%)**
  - Up to 3 even bi-lobar, all technically resectable in response or stable to chemotherapy (**new option added**) **(32%).**

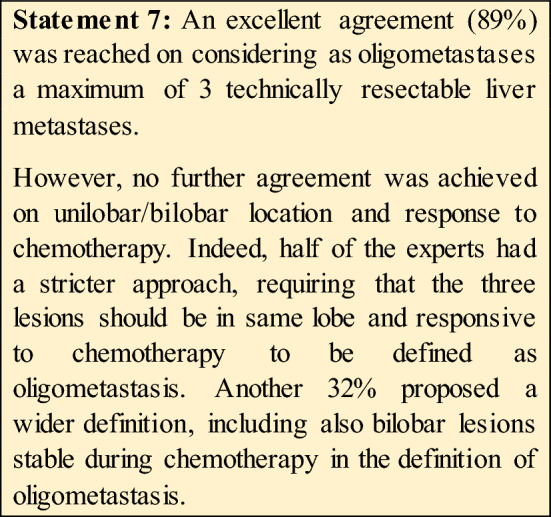



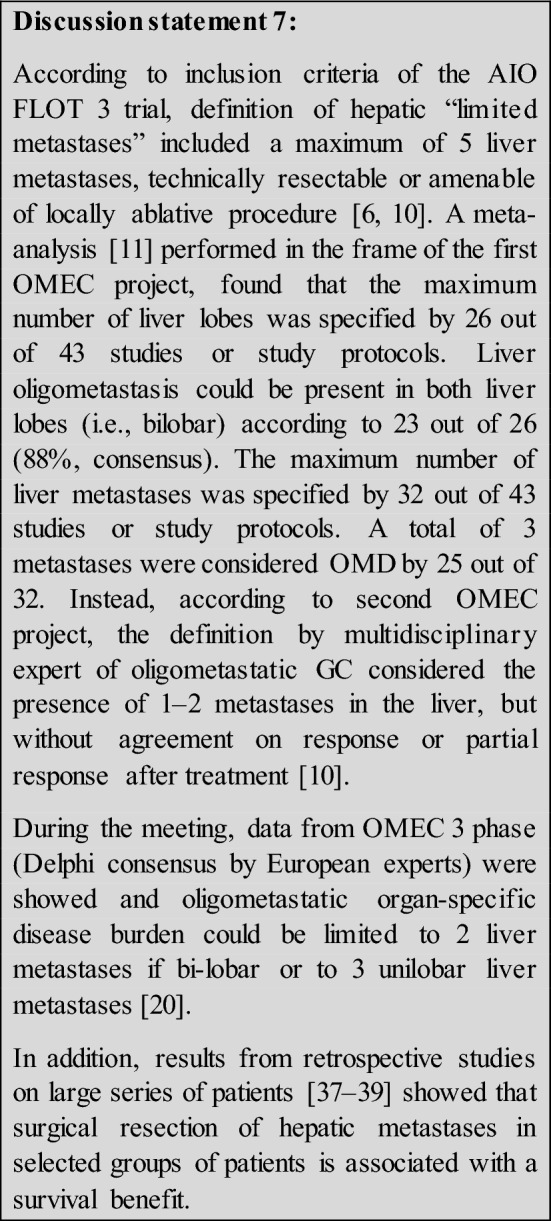

11. Which para-aortic lymph nodes stations do you include in the definition of oligometastatic GC? [Multiple choice is possible].
  - 16 a1 (aortic hiatus) **(6%)**
  - 16 a2 (from the origin of the celiac trunk to the lower margin of the left renal vein) **(16%)**
  - 16 b1 (from the inferior margin of the left renal vein to the superior margin of the inferior mesenteric artery) **(4%)**
  - 16 b2 (from the origin of the inferior mesenteric artery to the aortic bifurcation) **(2%)**
  - None of the above **(4%)**

A + B = **6%**

B + C = **22%**

A + B + C = **2%**

B + C + D = **2%**

A + B + C + D = **35%**

12. Which of the following conditions could be defined as oligometastatic GC with lymph node metastases? [Multiple choice is possible].
  - Clinically evident metastasis to para-aortic lymph-nodes in stations 16a2 and/or b1 **(4%)**
  - Clinically evident metastasis to para-aortic lymph-nodes in stations 16a1 and/or b2 **(2%)**
  - Clinically evident metastasis to other “posterior” stations (stations 12p, 13) **(4%)**
  - A combination of the previous, provided the response to chemotherapy **(72%)**
  - Clinically evident metastasis to para-aortic lymph-nodes in stations 16a2 and/or b1 provided the response to chemotherapy **(17%)**
13. Do you consider distant lymph nodes (e.g., supraclavear, mediastinic, other abdominal excluding paraortic in 16a2 and 16b1 stations) as oligometastatic disease?
  - Yes, in any case **(2%)**
  - Yes, if responsive to chemotherapy and technically resectable/amenable of local treatment **(11%)**
  - Yes, if technically resectable regardless response to chemotherapy **(0%)**
  - No, they should be considered as systemic metastatic disease in any cases, regardless response to chemotherapy and technical resectability **(87%)**

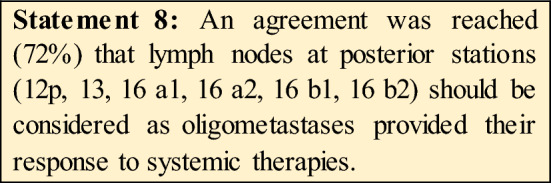



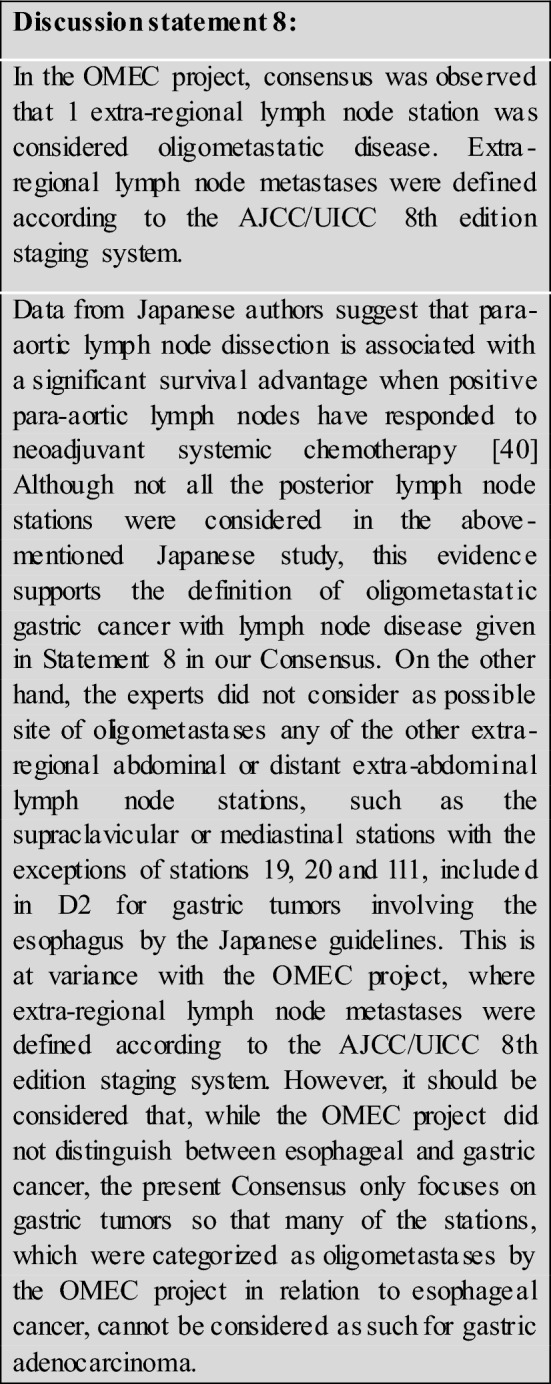

14. Does only positive cytology should be considered as oligometastatic disease?
  - Yes (31%)
  - No (**14%)**
  - Only if a conversion from positive to negative cytology after chemotherapy is documented **(55%)**
15. Does oligometastatic peritoneal gastric cancer exist?
  - Yes (specify the PCI cut off ….) **(91%).**
  - No (9%).

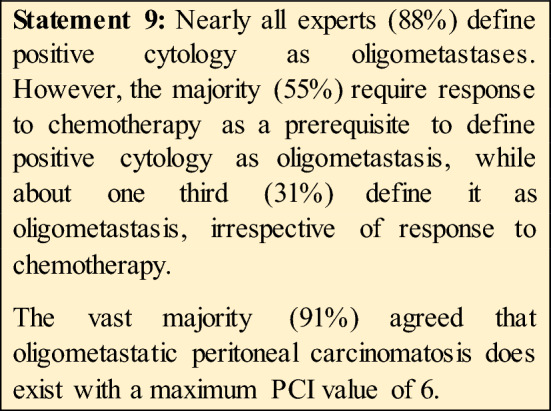



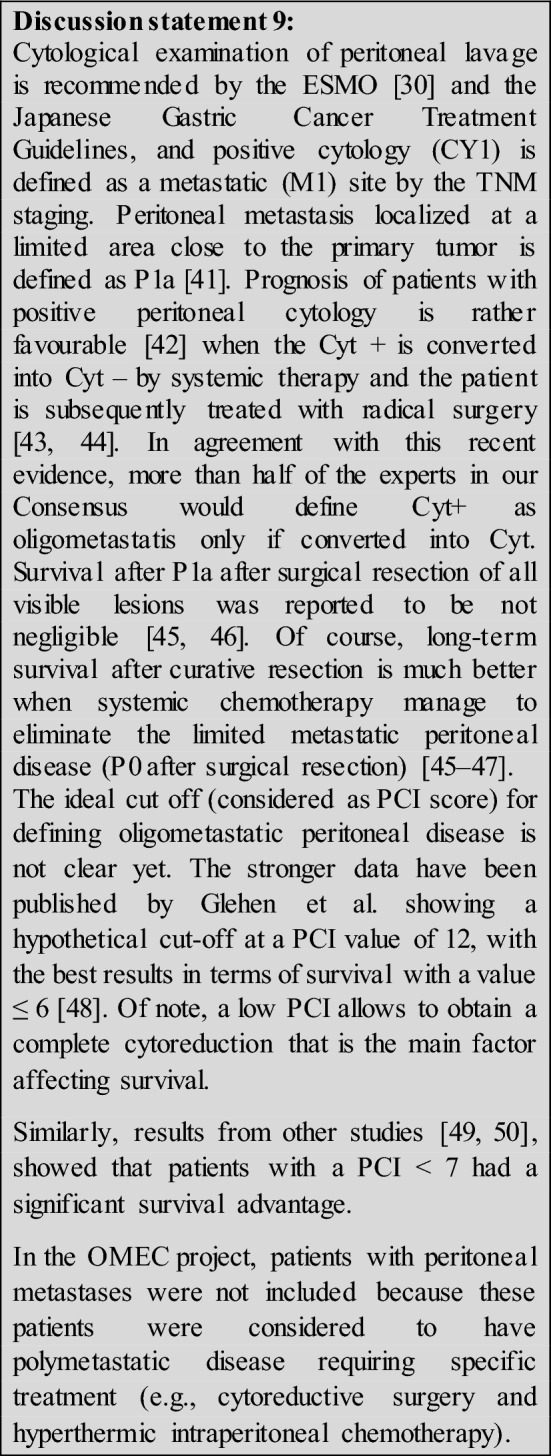

16. Do you think lung metastases could be considered as oligometastatic?
  - According to OMEC project, only 3 unilateral or 2 bilateral lesions **(82%).**
  - b. Yes, regardless the number and dimensions of lesions **(7%).**
  - No, in any case **(11%).**

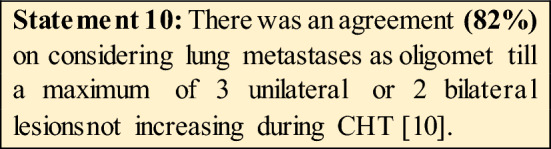

17. Do you think bone metastases could be included in oligometastatic disease?
  - No, in any cases **(56%)**
  - Yes, in all cases **(4%)**
  - Yes, only if 1 or 2 lesions provided the stability during chemo and amenable of local treatment **(40%**)

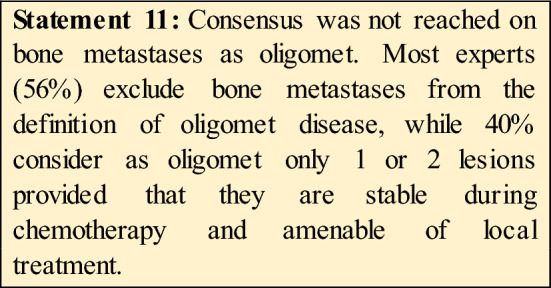



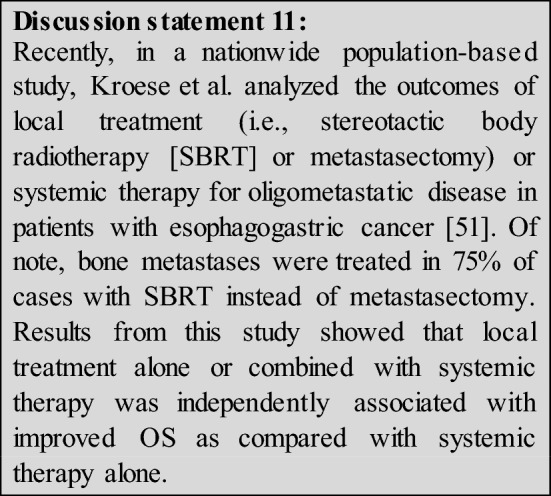

18. Do you believe that oligometastatic GC:
  - Should involve one site only (liver, lymph nodes, peritoneum)? **(53%)**
  - Can involve also more than one site **(32%)**
  - Can involve also more than one site provided that one of them is not peritoneum/Cyt + **(15%)**
19. **Do you believe that to define a metastatic GC as oligometastatic:**
  - There should be a response to chemotherapy both in terms of size and number of metastatic lesions **(34%)**
  - There should be a response to chemotherapy only in terms of number of metastatic lesions **(14%)**
  - The metastatic lesions can be in response or even stable to chemotherapy **(45%)**
  - The metastatic lesion can also progress to chemotherapy provided their technical resectability **(7%)**
  - Other (specify) **(0%.**

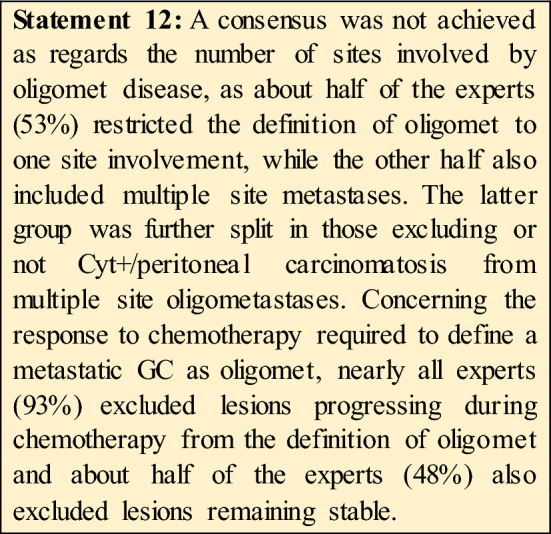



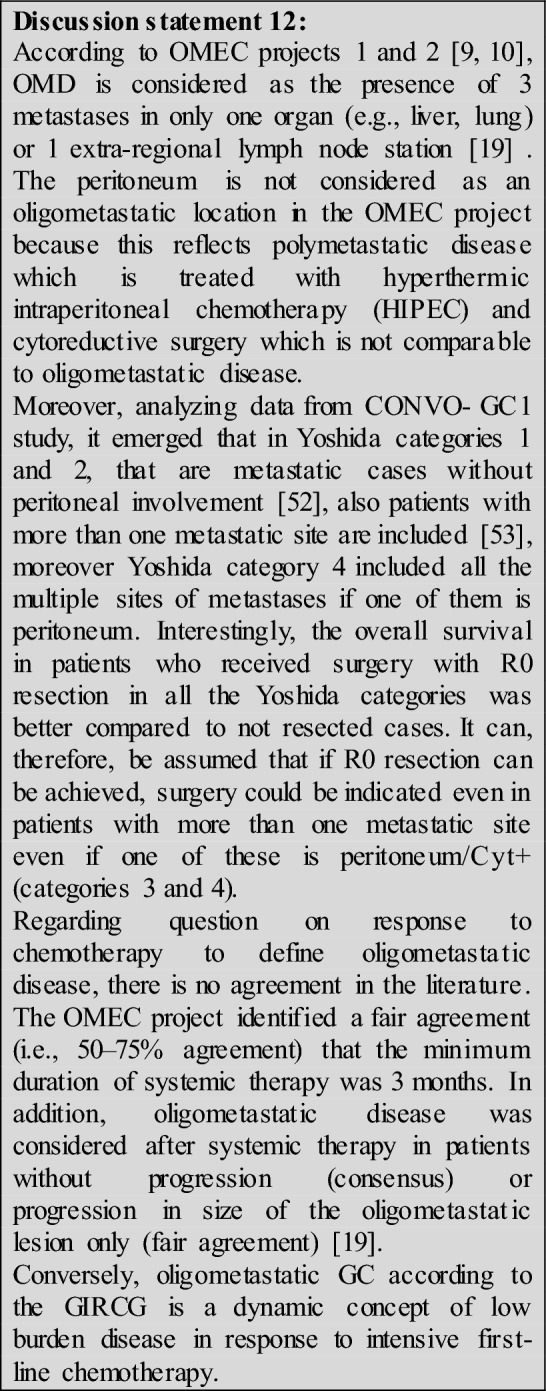

20 In case of oligometastatic HER2 negative GC, what kind of chemo do you suggest?
  - Standard first-line chemotherapy (FOLFOX) **(9%)**
  - b. FLOT **(23%)**
  - Immunotherapy OR chemo-immunotherapy in presence of biomarkers predictive of response **(63%)**
  - Other, please specify **(5%)**

21. In case of oligometastatic HER 2 positive GC, what kind of chemo do you suggest?
    - Platinum-based chemotherapy + trastuzumab **(86%)**
    - Chemotherapy + trastuzumab + immunotherapy **(14%)**
    - Other, please specify **(0%)**

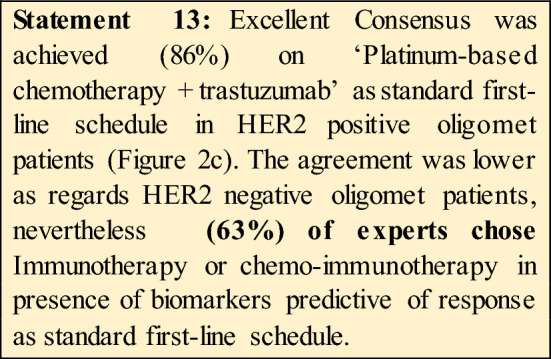



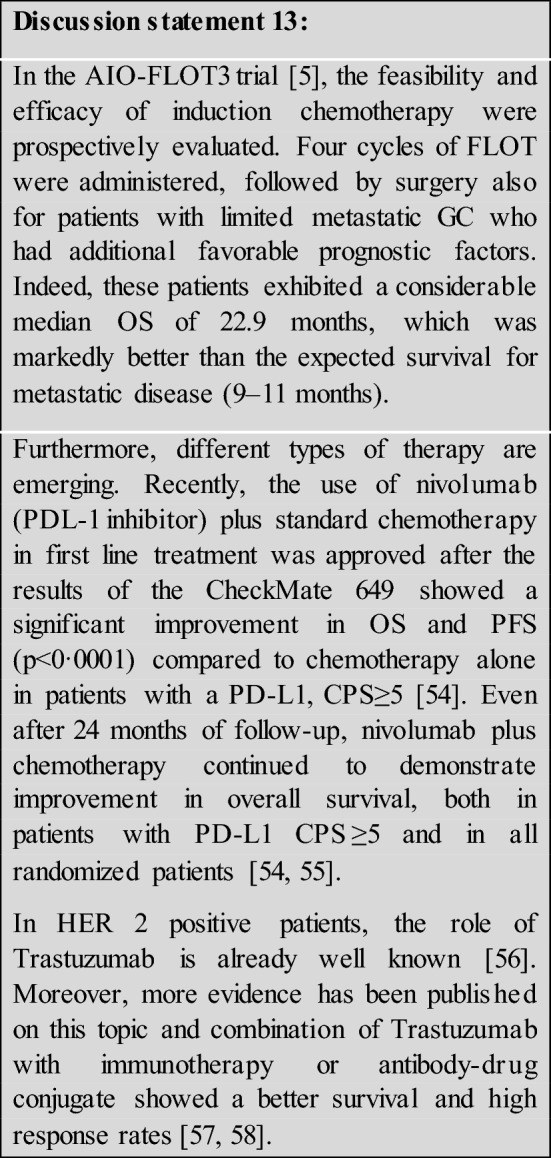

22. Which is the surgical indication in Oligometastatic GC (excluding peritoneum) after first-line treatment?
  - Radical intent surgery based on the initial volume of disease if stable or in response to chemotherapy **(75%)**
  - Radical intent surgery based on the initial volume of disease regardless of the degree of response to chemotherapy provided technical resectability **(20%)**
  - Other, please specify **(5%)**

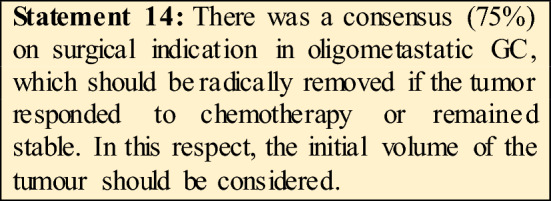



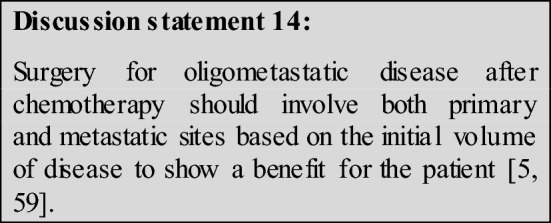

23. In case of positive cytology when do you consider surgery with radical intent?
  - Never **(0%)**
  - After first line chemotherapy, regardless it is converted into negative without HIPEC **(27%)**
  - After first line chemotherapy, regardless it is converted into negative with HIPEC **(38%)**
  - After first line chemotherapy, only in case of its conversion into negative, with HIPEC **(22%)**
  - After first line chemotherapy, only in case of its conversion into negative, without HIPEC **(14%)**
24. In case of surgery for Oligometastatic Peritoneal GC do you consider: [Multiple choice is possible].
  - Complete cytoreductive surgery including all the sites of positive biopsies at baseline and considering HIPEC not necessary **(21%)**
  - Complete cytoreductive surgery all the sites of positive biopsies at baseline and considering HIPEC necessary **(47%)**
  - Complete cytoreductive surgery according to residual disease without HIPEC **(8%)**
  - Complete cytoreductive surgery according to residual disease with HIPEC **(16%)**
  - Other, please specify **(8%)**

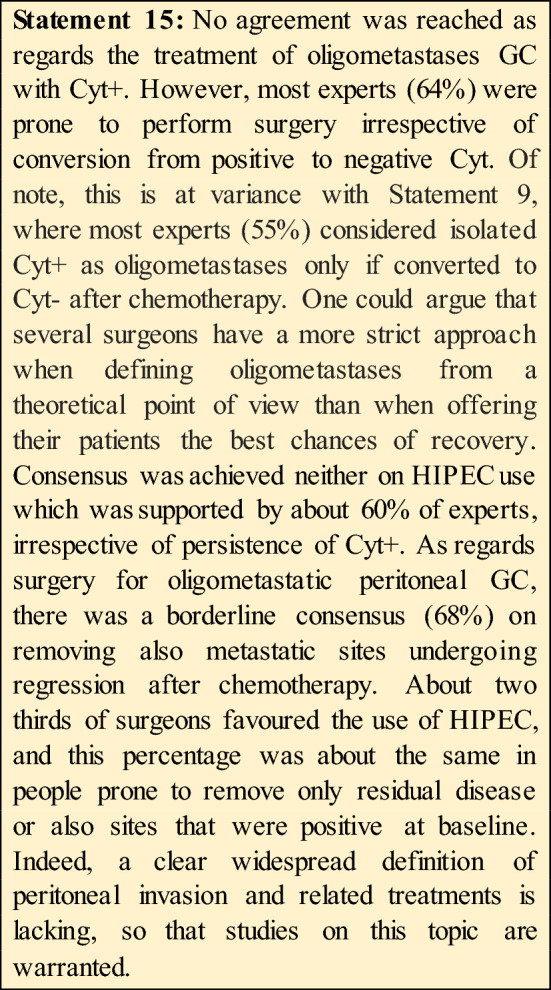



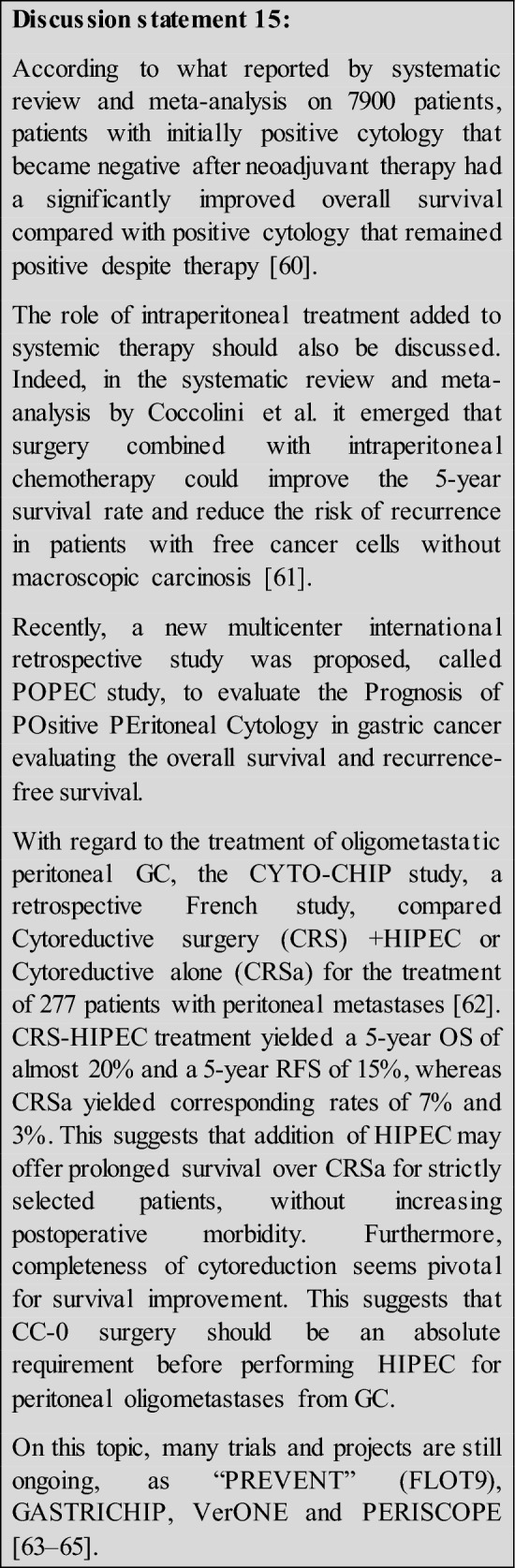

25. Rank the following sites from the most to the least consistent with the definition of Oligometastatic GC: Select the most suitable.
  - Nodal metastases **(69%)**
  - Liver metastases **(14%)**
  - Peritoneal metastases **(0%)**
  - Positive cytology **(14%)**
  - Other, please specify **(3%)**

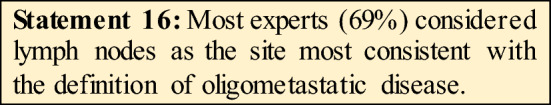



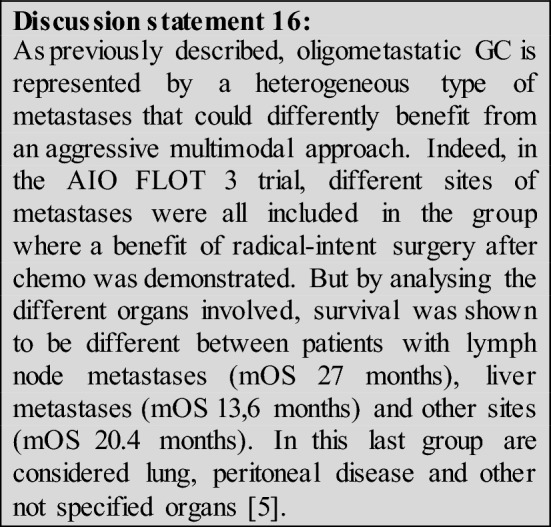

26. Which is your definition of conversion surgery?
  - Surgery on the residual disease after chemotherapy in case metastatic GC which was initially not resectable for technical and/or oncological reasons but nevertheless responded to first-line treatment **(98%)**
  - Surgery on the residual disease after chemotherapy in case metastatic GC which was initially not resectable for technical and/or oncological reasons regardless the response to first-line treatment **(2%)**
  - Surgery on the residual disease after chemotherapy in case metastatic GC which was technically and/or oncologically resectable at the time of diagnosis, regardless the response to first-line treatment **(0%)**

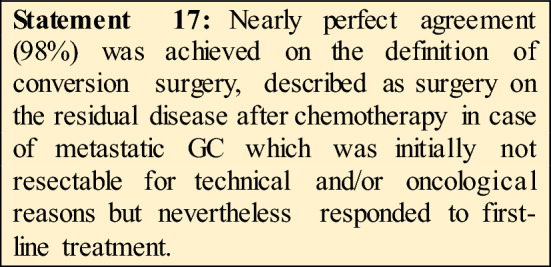



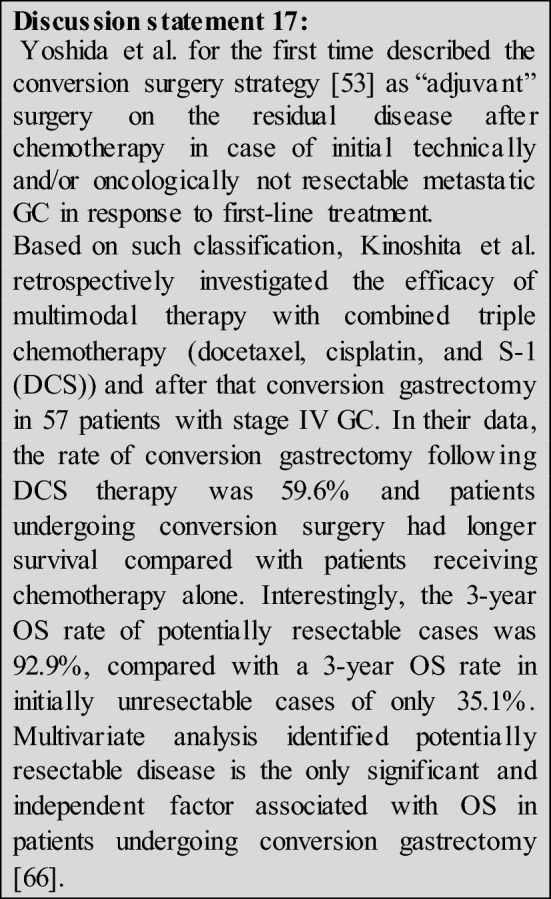

27. How do you manage the site that was responsible for initial unresectability in case of its clinical complete response?
  - Try to resect it **(74%)**
  - b. Never resect it **(21%)**
  - No resection, but place markers for subsequent possible radiotherapy **(5%)**
28. How do you consider the case of the previous question?
  - R0 (97%)
  - R1 **(3%)**
  - R2 (0%)

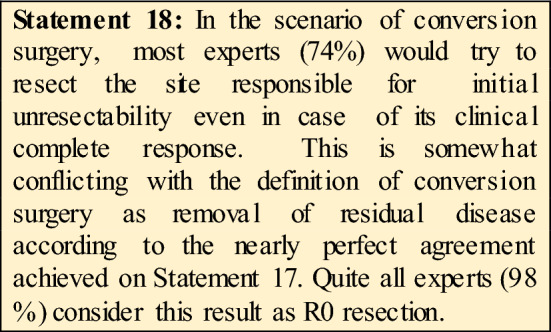

29. Do you think that conversion surgery could have the same benefits if first-line treatments did include the use of immunotherapy?
  - Yes **(18%)**
  - b. No **(5%)**
  - No Data Available **(77%)**

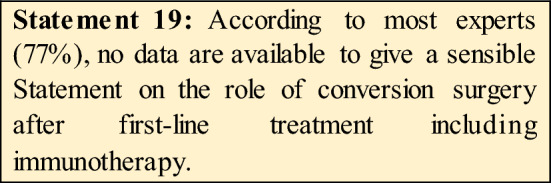



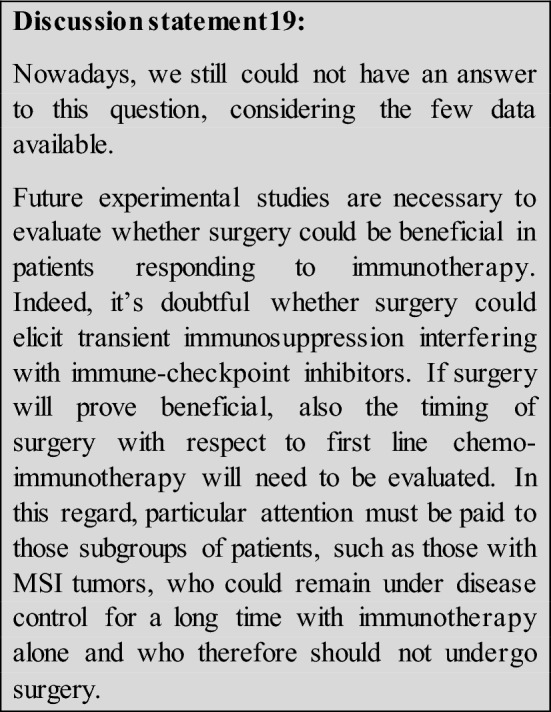


### (C) How to care

30. Do you have the possibility of palliative team in your centre?
  - Yes **(97%)**
  - No **(3%)**
31. When do you stop oncologic palliative treatments?
  - On patient’s request **(6%)**
  - bIn relation to performance status **(6%)**
  - In case of multiple metastatic sites **(0%)**
  - After progression despite of oncological treatments **(26%)**
  - Shared decision (**new option added**) **(63%)**
32. What is the indication for nutritional support in end-stage patients?
  - Always **(12%)**
  - Never **(6%)**
  - In relation to patient’s general condition and life-expectancy **(74%)**
  - On patient’s request **(9%)**

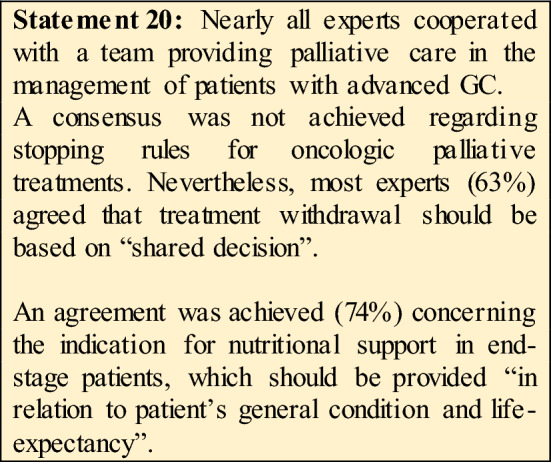


## Discussion

This is the first multidisciplinary consensus on metastatic gastric cancer covering all aspects that guide clinical management of the patients.

In recent years, novel and more effective oncological treatments for stage IV GC improved the prognosis or, in some individuals, led to cure. According to the AIO-FLOT3 trial [5], patients with “oligometastatic” GC which is defined as a low disease burden stage IV GCs may benefit from combined approaches including surgery. Although there are shared ideas about this concept, it remains difficult in clinical practice to identify patients with oligometastatic GC and who may benefit from more aggressive treatment. There are some papers aimed at creating consensus regarding the definition of oligometastases including the initiative by the European OMEC group. However, there is still no universal agreement on some issues. Furthermore, unlike other studies, this one focus only on stomach tumors (including Siewert 3), excluding tumors of the esophagus and EGJ which have different biological characteristics.

Furthermore, metastatic GC is often not oligometastatic. Many patients have an extensive burden of the disease at diagnosis and there are technical and/or oncological reasons that do not allow radical treatment including surgery. However, effective systemic treatment may move the patients towards conversion surgery aimed at removing residual tumor. But there are no universally agreed strategies for this.

**Fig. 1:**
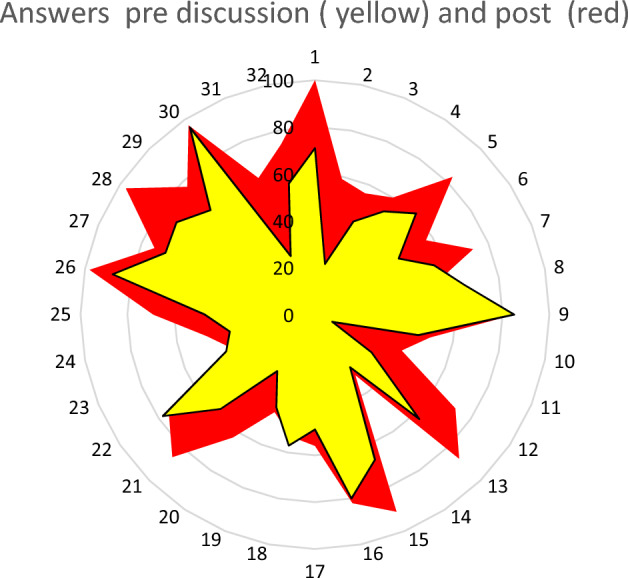
Overview of issues before and after consensus

The Workshop led to a better agreement among experts on several issues as shown in the Fig. 1. The agreement was reached on items related to diagnosis and staging, definition and anatomical details of oligometastatic GC, the concept behind possible surgical indications for stage IV GC.

Currently, CT scan is considered the best tool for staging GC, but this requires skilled radiologist. A more accurate dimensional cut-off for lymph node staging is also required. CT scan is the preferred method even for restaging GC, RECIST criteria are used to evaluate the regression after systemic treatments, but other information should be added to RECIST such as radiomics findings.

Of note, there was an agreement on performing staging laparoscopy in locally advanced GC irrespective of tumor histology. However, there are no standard protocols for staging laparoscopy neither for the evaluation of peritoneal cytology. Accordingly, during the Workshop, a specific project was proposed to fill this gap.

We had Consensus that to date, the following biomarkers should be evaluated on primary tumor biopsies at time of diagnosis in all the newly detected metastatic GC: HER2, PDL1 (CPS), MSI, EBV. However, guidelines on gastric cancer biomarkers should be tailored to different geographic areas, to take into account differences between Eastern Asia and Europe/North America.

Moreover, it should be noted that, since the questionnaire for this survey was designed, the results of two recent studies have been published documenting the effectiveness of the association of an anti-Claudin 18 antibody with chemotherapy in metastatic gastric cancer [67, 68]. Therefore, even if not yet approved in clinical practice, it is likely that this association will soon be the first-line option of choice in patients with Claudin 18 positive stage IV GC and that, therefore, the IHC evaluation for Claudin 18 must be included in the panel of standard biomarkers to be evaluated in all cases of newly diagnosed metastatic gastric cancer. Experts have not reached a consensus regarding the need to biopsy the metastatic site. Unfortunately, this need was discussed only to characterize the possible heterogeneity of predictive biomarkers between primary tumor and metastases, while the usefulness of biopsy in confirming the metastatic nature of distant lesions, especially for peritoneal lesions, was overlooked.

**Fig. 2:**
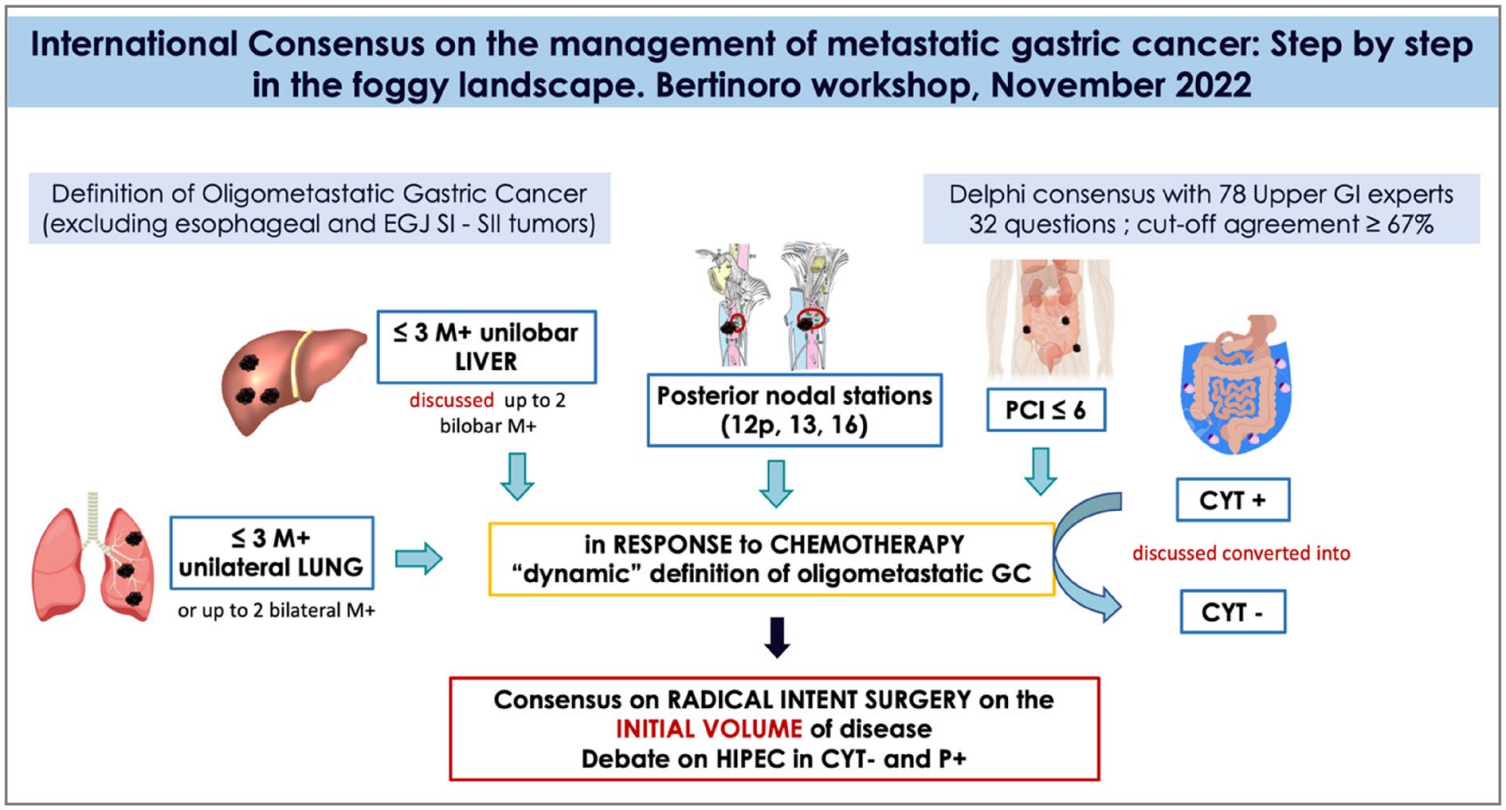
Visual abstract of consensus main finding on oligometastatic GC

Regarding the definition of oligometastatic for the various sites, we agreed that (Fig. 2).

for the liver there is agreement on the maximum number of metastases which is 3, but then the experts are divided as to the distribution (uni- versus bilobular). Distant lymph nodes are considered as oligometastatic if the stations 16a2, 16b1 are involved. Other “posterior stations” (12p, 13, 16a1, 16b1) could be included in the oligometastatic definition provided their response to systemic treatments.

An important finding of our Consensus is the agreement on oligometastatic peritoneal disease and peritoneal cytology. Indeed, in other Consensus [10] peritoneal cytology and/or metastases have been excluded from oligometastatic setting as it is considered as an incurable condition. More in detail, in our Consensus, a low burden peritoneal disease (PCI up to 6) is considered as oligometastatic GC.

For Cyt + there was not a clear agreement among all the experts, as some consider Cyt as oligomet only if it is converted from Cyt + to Cyt− after systemic treatment, while others always considered it ad oligometasis. However, by taking together all the expert’s answers, there is a consensus in considering Cyt+ as oligomet when it has been converted by chemotherapy.

Another important result of the present consensus is that a disease can be defined as oligometastatic only after evaluating its response to systemic therapies. Indeed, it would be important to have biomarkers/predictors to select those cases of metastatic gastric cancer, who could benefit from aggressive multimodal treatment. However, such biomarkers/predictors are still lacking, to a large extent. Hence, response to chemotherapy is a good indicator to guide treatment choice. This “dynamic” definition of the concept of oligometastases is a relevant achievement of the present Experts Consesus.

Then the experts discussed the optimal first-line schedule in oligometastatic patients. Excellent agreement was achieved (86%) on ‘Platinum- based chemotherapy + trastuzumab’ as standard first-line schedule in HER2 positive cases. Of note, the KEYNOTE-811 study, a randomized phase III trial, showed a superior objective response with Pembrolizumab compared with placebo when added to Trastuzumab plus fluoropyrimidine and platinum-based chemotherapy as first-line treatment in patients with locally advanced or metastatic HER2-positive gastro-oesophageal junction carcinoma [57]. These results were recently confirmed also by a third interim analysis (with median FU of 38.4 months) of the study on 698 patients, in which both disease free survival (10.0 months versus 8.1 months (7.1–8.6; HR 0·73 [0.61–0.87]) and overall survival (20.0 months versus 16.8 months; HR 0.84 [0.70–1.01]) were significantly higher in patients treated with Pembrolizumab compared to placebo. This advantage is higher specifically in patients with tumour with PD-L1 combined positive score of 1 or more [69]. Accordingly, the EMA released the following Statement on August 2023: “KEYTRUDA, in combination with trastuzumab, fluoropyrimidine and platinum-containing chemotherapy, is indicated for the first-line treatment of locally advanced unresectable or metastatic HER2-positive gastric or gastro-oesophageal junction adenocarcinoma in adults whose tumours express PD-L1 with a CPS ≥ 1”. However, this practice still needs to be implemented in several countries.

The agreement on the optimal first line treatment in HER2 negative oligometastatic patients, was lower, nevertheless (63%) of experts chose “immunotherapy or chemo-immunotherapy in presence of biomarkers predictive of response” as standard first-line schedule. Results of the KEYNOTE-859 study, a randomized phase III trial, designed for patients with Her 2 negative advanced unresectable or metastatic gastric/gastroesophageal junction carcinoma confirm this approach: patients treated with Pembrolizumab plus chemotherapy had a significant and clinically meaningful improvement in overall survival with manageable toxicity compared to participants treated with placebo plus chemotherapy (median overall survival of 12·9 months in Pembrolizumab [95% CI 11.9–14.0] vs 11·5 months in placebo group [10.6–12.1]; hazard ratio [HR] 0.78 [95% CI 0.70–0.87]; *p* < 0.0001).

The advantage on overall survival is higher in patients with tumours with PD-L1 CPS of 10 or higher [70].

Unfortunately, whether the best chemotherapy regimen in these HER2 negative oligometastatic patients, in ideal contitions, is FLOT or FOLFOX has not been discussed in the present Consensus, but it remains an important point of debate in clinical practice. Furthermore, in the meantime, the results of a new trial were presented at ESMO2023 [71] showing that a modified FLOT scheme (mFLOT/TFOX) is more effective than oxaliplatin and 5-fluorouracil (FOLFOX) for patients with metastatic/unresectable gastric or gastroesophageal junction (GEJ) adenocarcinoma.

Furthermore, we aimed to clarify the possible surgical indication in stage IV gastric cancer. Of note, we had a good agreement on the planned extent of surgery within the oligometastatic concept that should be a radical-intent surgery on the initial volume of disease, as assessed at the time of diagnosis.

On the contrary, there are other cases with extensive, non-oligometastatic disease, in such cases, “conversion” surgery and/or ablative treatments should be a radical-intent procedure on the residual volume of disease after an exceptional response to systemic treatments.

Of note, even though there was a nearly perfect agreement on the definition of conversion surgery (“surgery on the residual disease after chemotherapy in case of metastatic GC which was initially not resectable for technical and/or oncological reasons but nevertheless responded to first-line treatment” nevertheless, in the subsequent questions there was consensus on the attempt to surgically resect the site responsible for initial unresectability even in case of its clinical complete response. This indicates that there is no clarity on some concepts in clinical practice.

It should be noted that the role of local ablation treatments other than surgery has been taken into consideration much more in the context of advanced metastatic than oligometastatic disease. Indeed, although in some oligometastatic cases with localizations difficult to surgically excise, other local ablative treatments can also be chosen with curative intent, the first-choice treatment remains surgery. As underlined several times during the plenary meeting, one of the objectives of the present Consensus was to identify the anatomical definitions of oligometastatic disease that were amenable to local and in particular to surgical treatment for gastric cancer only, including Siewert 3 but not the other cardia adenocarcinomas or esophageal tumors. This marks a difference by the OMEC project, which considered also the latter locations. For instance, an isolated extraregional metastasis involving supraclavicular lymph nodes could be considered as oligometastatic disease for all cancers considered by the OMEC project (including gastric cancer) but not according to the present Consensus. Thus, the present consensus allows us to offer a vision that is not antithetical to OMEC, but complementary to it and of practical interest for the gastric surgeon.

The results of the eastern multicenter trial CONVO-GC-1[53] are among the most relevant pieces of evidence on the topic of metastatic GC. In the latter study metastatic GC was classified according to Yoshida [52]: category 1 (technically resectable, as solitary liver metastases < 5 cm, para- aortic lymphnode 16 a2/b1 or only positive peritoneal cytology) could be considered as oligometastatic disease, while in all the other categories (2–4) surgery was regarded as conversion surgery. Looking at radicality after surgery, R0 resections rates were higher in category 1 and 2 (about 75%) and progressively lower in categories 3 and 4 (59% and 56.4%, respectively). Of note, the median survival time (MST) was significantly longer in patients who underwent R0 resections. Interestingly, the MST in category 1 was not superior to that of other categories (47.8 months vs 116.7 months vs 44.8 months vs median not reached, respectively for category 1, 2, 3 and 4).

These results are interesting and bring new hope to patients with metastatic gastric cancer. A still open question is whether to remove the initial cancer volume or only residual volume after chemotherapy. The best surgical approach should be established thorough comparison of Eastern and Western series, followed by a randomized clinical trial on this specific problem.

Finally, experts agreed that patients’ nutritional and psychologic support may improve the opportunities to propose new treatment in compromised situations, but ethics aspects, patient’s respect, and human approach to the end of the life on metastatic non responders, must never be forgotten. The limitations of this manuscript are mainly related to the lack of a degree of evidence and the strength of the recommendations as the methodology used and the relevance of the studies currently present in the literature did not allow us to reach these. However, the representativeness of the participants and the extent of the topics covered allowed us to provide a first practical guidelines in this emerging scenario.

The future in this field is undoubtedly the advancement of knowledge in the translational field on the complex interplay between tumor, tumor microenvironment and how this impacts the response to treatments. The design of innovative trials on this basis will open new horizons and new treatment possibilities for this lethal condition.

### Supplementary Information

Below is the link to the electronic supplementary material.

Supplementary file1 (DOCX 28 KB)
