# Correction: International consensus on the management of metastatic gastric cancer: step by step in the foggy landscape

**DOI:** 10.1007/s10120-024-01520-7

**Published:** 2024-06-17

**Authors:** Paolo Morgagni, Maria Bencivenga, Fatima Carneiro, Stefano Cascinu, Sarah Derks, Maria Di Bartolomeo, Claire Donohoe, Clarisse Eveno, Suzanne Gisbertz, Peter Grimminger, Ines Gockel, Heike Grabsch, Paulo Kassab, Rupert Langer, Sara Lonardi, Marco Maltoni, Sheraz Markar, Markus Moehler, Daniele Marrelli, Maria Antonietta Mazzei, Davide Melisi, Carlo Milandri, Paul Stefan Moenig, Bianca Mostert, Gianni Mura, Wojciech Polkowski, John Reynolds, Luca Saragoni, Mark I. Van Berge Henegouwen, Richard Van Hillegersberg, Michael Vieth, Giuseppe Verlato, Lorena Torroni, Bas Wijnhoven, Guido Alberto Massimo Tiberio, Han-Kwang Yang, Franco Roviello, Giovanni de Manzoni, Bertinoro Workshop Working Group, William Allum, Giulio Bagnacci, Gian Luca Baiocchi, Felix Berlth, Laura Borgno, Jimmy So Bok Yan, Riccardo Caccialanza, Francesco Casella, Claudia Castelli, Mikael Chevallay, Simona Corso, Paulo Matos Da Costa, Mariagiulia Dal Cero, Maurizio De Giuli, Stefano De Pascale, Annibale Donini, Domenico D’Ugo, Giorgio Ercolani, Federica Filippini, Massimo Framarini, Ewelina Frejlich, Uberto Fumagalli Romario, Simone Giacopuzzi, Silvia Giordano, Luigina Graziosi, Henk Hartgrink, Arnulf H. Hölscher, Jeesun Kim, Tiuri Kroese, Lourenco Laercio Gomes, Lucio Lara Santos, Drolaiz Liu, Florian Lordick, Luigi Marano, Elisabetta Marino, Giovanni Martinelli, Hans-Joachim Meyer, Silvia Ministrini, Paul F. Mansfield, Chiara Molinari, Manlio Monti, Yusef Moulla, Magnus Nilsson, Sara Patuzzo, Manuel Pera, Osvaldo Antonio Prado Castro, Alberto Quinzii, Ilario Giovanni Rapposelli, Stefano Rausei, Rossella Reddavid, Fausto Rosa, Riccardo Rosati, Romina Rossi, Giandomenico Roviello, Julia Rudno-Rudzinska, Michele Sacco, Massimiliano Salati, Paul M. Schneider, Leonardo Solaini, Masanori Terashima, Anna Tomezzoli, Martina Valgiusti, Antonio Carlos Weston, Kielan Wojciech, Goetze Thortsen

**Affiliations:** 1 Department of General Surgery, Morgagni-Pierantoni Hospital, Forlì, Italy; 2 General and Upper GI Surgery, Department of Surgery, University Hospital Verona, University of Verona, Verona, Italy; 3 Department of Pathology, Centro Hospitalar de São João, Institute of Molecular Pathology and Immunology of the University of Porto (Ipatimup), Porto, Portugal; 4 Department of Medical Oncology, Comprehensive Cancer Center, Università Vita-Salute, IRCCS Ospedale San Raffaele, Milan, Italy; 5 Department of Medical Oncology, Amsterdam UMC, Vrije Universiteit Amsterdam, Amsterdam, The Netherlands; 6 Department of Medical Oncology, Fondazione IRCCS Istituto Nazionale dei Tumori, Milan, Italy; 7 Medicinal Chemistry, Trinity Translational Medicine Institute, Trinity Centre for Health Sciences, Trinity College Dublin, The University of Dublin, St. James’s Hospital, Dublin 8, Ireland; 8 Department of Digestive and Oncologic Surgery, Claude Huriez University Hospital, Centre Hospitalier Universitaire (CHU) Lille, Université de Lille, Lille, France; 9 Department of Surgery, Cancer Center Amsterdam, Amsterdam UMC, University of Amsterdam, Amsterdam, The Netherlands; 10 Department of General, Visceral and Transplant Surgery, University Medical Center, University of Mainz, Mainz, Germany; 11 Department of Visceral, Transplant, Thoracic and Vascular Surgery, University Hospital of Leipzig, Leipzig, Germany; 12 Department of Pathology, GROW School for Oncology and Developmental Biology, Maastricht University Medical Center, Maastricht, The Netherlands; 13 Pathology and Data Analytics, Leeds Institute of Medical Research at St James’s, University of Leeds, Leeds, United Kingdom; 14 Gastric Surgery Division, BP Gastric Surgery Department, Santa Casa Medical School, São Paulo, Brazil; 15 Institute of Pathology and Microbiology, Johannes Kepler University Linz, Altenberger Strasse 69, 4040 Linz, Austria; 16 Istituto Oncologico Veneto IOV-IRCCS, Padua, Italy; 17 Unit of Palliative Care, Istituto Scientifico Romagnolo per lo Studio e la Cura dei Tumori (IRST) IRCCS, Meldola, Forlì-Cesena Italy; 18 Surgical Interventional Trials Unit, University of Oxford, Oxford, UK; 19 Department of Medicine, Johannes-Gutenberg University Clinic, Mainz, Germany; 20 Unit of General Surgery and Surgical Oncology, Department of Medicine Surgery and Neurosciences, University of Siena, 53100 Siena, Italy; 21 Unit of Diagnostic Imaging, Department of Medical, Surgical and Neuro Sciences and of Radiological Sciences, Azienda Ospedaliero-Universitaria Senese, University of Siena, 53100 Siena, Italy; 22 Medical Oncology at the Department of Medicine, University of Verona, Verona, Italy; 23 Department of Oncology, San Donato Hospital, 52100 Arezzo, Italy; 24 Surgery Department, Geneva University Hospitals, Geneva, Switzerland; 25 Department of Medical Oncology, Erasmus MC Cancer Institute, Dr. Molewaterplein 40, 3015 GD Rotterdam, The Netherlands; 26 Department of Surgery, San Donato Hospital, Arezzo, Italy; 27 Department of Surgical Oncology, Medical University of Lublin, Radziwiłłowska 13 St, 20-080 Lublin, Poland; 28 Trinity College, Dublin, Ireland; 29 Pathology Unit, Santa Maria delle Croci Ravenna Hospital, Ravenna, Italy; 30 University Medical Center Utrecht, Heidelberglaan 100, 3584 CX Utrecht, The Netherlands; 31 Institute of Pathology, Klinikum Bayreuth, Bayreuth, Germany; 32 Department of Diagnostics and Public Health, Section of Epidemiology and Medical Statistics, University of Verona, Verona, Italy; 33 Department of Surgery, Erasmus MC-University Medical Centre Rotterdam, Rotterdam, Netherlands; 34 Surgical Clinic, Department of Clinical and Experimental Sciences, University of Brescia, Brescia, Italy; 35 Surgical Department, SNUH National Cancer Center, Seoul, Korea

**Correction: Gastric Cancer** 10.1007/s10120-024-01479-5

In this article the collaborators group name “Bertinoro Workshop Working Group” was missing from the author list. The author name Heike Grabsch was incorrectly written as Heike Grabsh.

**Collaborators and Affiliations (Bertinoro Workshop Working Group)**

William Allum^36^, Giulio Bagnacci^21^, Gian Luca Baiocchi^37^, Felix Berlth^10^, Laura Borgno^38,39,40^, Jimmy So Bok Yan^41^, Riccardo Caccialanza^42^,Francesco Casella^2^, Claudia Castelli^43^, Mikael Chevallay^24^, Simona Corso^44^, Paulo Matos Da Costa^45^, Mariagiulia Dal Cero^46^, Maurizio De Giuli^47^, Stefano De Pascale^48^, Annibale Donini^49^, Domenico D’Ugo^50^, Giorgio Ercolani^51^, Federica Filippini^2^, Massimo Framarini^1^, Ewelina Frejlich^52^, Uberto Fumagalli Romario^48^, Simone Giacopuzzi^2^, Silvia Giordano^44^, Luigina Graziosi^49^, Henk Hartgrink^53^, Arnulf H. Hölscher^54^, Jeesun Kim^35^, Tiuri Kroese^55^, Lourenco Laercio Gomes^56^, Lucio Lara Santos^57^, Drolaiz Liu^58^, Florian Lordick^59^, Luigi Marano^20^, Elisabetta Marino^49^, Giovanni Martinelli^60^, Hans-Joachim Meyer^61^, Silvia Ministrini^34^, Paul F. Mansfield^62^, Chiara Molinari^60^, Manlio Monti^60^, Yusef Moulla^63^, Magnus Nilsson^64^, Sara Patuzzo^65^, Manuel Pera^46^, Osvaldo Antonio Prado Castro^14^, Alberto Quinzii^22^, Ilario Giovanni Rapposelli^60^, Stefano Rausei^66^, Rossella Reddavid^47^, Fausto Rosa^67^, Riccardo Rosati^68^, Romina Rossi^17^, Giandomenico Roviello^69^, Julia Rudno-Rudzinska^52^, Michele Sacco^2^, Massimiliano Salati^70^, Paul M Schneider^71^, Leonardo Solaini^51^, Masanori Terashima^72^, Anna Tomezzoli^43^, Martina Valgiusti^60^, Antonio Carlos Weston^73^, Kielan Wojciech^52^, Goetze Thortsen^74^

^36^Department of Surgery, Royal Marsden Hospital, London, UK

^37^Department of Clinical and Experimental Sciences, University of Brescia, Brescia, Italy

^38^Chief of Surgery Department of National Cancer Institute ASSE, Uruguay

^39^Chief of Surgery Department Las Piedras Hospital ASSE, Uruguay

^40^Ex Associate Professor of General Surgery Medicine Faculty Udelar, Uruguay

^41^Department of Surgery, National University Hospital, Yong Loo Lin School of Medicine, National University of Singapore, Singapore

^42^Clinical Nutrition and Dietetics Unit, Fondazione IRCCS Policlinico San Matteo, 27100 Pavia, Italy

^43^Section of Pathology, Department of Diagnostics and Public Health University of Verona, Verona, Italy

^44^Department of Oncology, Torino University, Candiolo Cancer Institute-FPO, IRCCS Italy

^45^Departamento de Cirurgia, Faculdade de Medicina, Universidade de Lisboa, Lisboa, Portugal

^46^Section of Gastrointestinal Surgery, Hospital Universitario del Mar, Hospital del Mar Medical Research Institute (IMIM). Department of Surgery, Universitat Autònoma de Barcelona, Barcelona, Spain

^47^Department of Oncology, Surgical Oncology and Digestive Surgery Unit, San Luigi University Hospital, University of Turin, Orbassano, Turin, Italy

^48^Department of Digestive Surgery, European Institute of Oncology, Istituto di Ricovero e Cura a Carattere Scientifico (IRCCS), 20141 Milan, Italy

^49^General and Emergency Surgery, University of Perugia, Perugia, Italy

^50^Fondazione Policlinico Universitario Agostino Gemelli IRCCS, Rome, Italy

^51^Department of Medical and Surgical Sciences (DIMEC), University of Bologna, Morgagni-Pierantoni Hospital, Forlì, Italy

^52^2nd Department of General and Oncological Surgery, Wroclaw Medical University Hospital, Wrocław, Poland

^53^Department of Surgery, Leiden University Medical Center, Leiden, the Netherlands

^54^Head Contilia Center for Esophageal Diseases Elisabeth Hospital Essen, Cooperation Partner of West German Tumor Center, University Medicine Essen, Germany

^55^Department of Radiation Oncology, University Hospital Zurich, University of Zurich, Zurich, Switzerland

^56^Federal University of São Paulo, São Paulo, SP, Brazil

^57^Surgical Oncology Department, Portuguese Oncology Institute of Porto (IPO-Porto), 4200-072 Porto, Portugal

^58^Department of Pathology, GROW School for Oncology and Reproduction, Maastricht University Medical Center, Maastricht, the Netherlands

^59^Department of Medical Oncology, University Cancer Center Leipzig, Leipzig University Medical Center, Leipzig, Germany

^60^IRCCS Istituto Romagnolo per Lo Studio Dei Tumori (IRST) "Dino Amadori", Meldola, Italy

^61^German Society of Surgery, Berlin, Germany

^62^Department of Surgical Oncology, UT MD Anderson Cancer Center, Houston, Texas, USA

^63^Department of Visceral, Transplant, Thoracic and Vascular Surgery, University Hospital of Leipzig AöR, Leipzig, Germany

^64^Division of Surgery, Department of Clinical Science, Intervention and Technology, Karolinska Institute, Stockholm, Sweden; Department of Upper Abdominal Diseases, Karolinska University Hospital, Stockholm, Sweden

^65^School of Medicine and Surgery, University of Verona, 37134, Verona, Italy

^66^Depertment of Surgery, Ospedale S. Antonio Abate, ASST Valle Olona, Gallarate, Italy

^67^Digestive Surgery Unit, Fondazione Policlinico Universitario Agostino Gemelli IRCCS, Rome, Italy

^68^Department of Gastrointestinal Surgery, IRCCS-San Raffaele Research Hospital, Milan, Italy

^69^Department of Health Sciences, Section of Clinical Pharmacology and Oncology, University of Florence, Viale Pieraccini, 6, 50139, Florence, Italy

^70^Division of Oncology, Department of Oncology and Hematology, University Hospital of Modena, 41121 Modena, Italy

^71^Digestive Oncology Tumor Center, Hirslanden Medical Center, Zurich, Switzerland

^72^Department of Gastric Surgery, Shizuoka Cancer Center, Shizuoka, Japan

^73^Chef do Serviço de Cirurgia Geral da Santa Casa, Diretor Médico da Ingastro, Department of Surgery, Porto Alegre, Brazil

^74^Institute of Clinical Cancer Research, Krankenhaus Nordwest, UCT-University Cancer Center, Frankfurt, Germany; IKF Klinische Krebsforschung GmbH am Krankenhaus Nordwest, Frankfurt, Germany

The original article has been corrected.

