# Supplementary material for: International consensus on the management of metastatic gastric cancer: step by step in the foggy landscape: Bertinoro Workshop, November 2022
Source: Gastric Cancer. 2024 Apr 18;27(4):649–71. doi: 10.1007/s10120-024-01479-5 (PMC11193703; doi:10.1007/s10120-024-01479-5)
Supplement: Supplementary file 1 — Supplementary file1 (DOCX 28 KB) [file 10120_2024_1479_MOESM1_ESM.docx]

**SUPPLEMENTARY TABLE**

**Supplementary table 1.** Nominatives of the Expanded Working Group involved in the rating process and who have agreed to participate.

| **SURNAME NAME** | **COUNTRY** | **SPECIALTY** | **FIRST ROUND** | **SECOND ROUND** |
| --- | --- | --- | --- | --- |
|  |  |  |  |  |
| 1. ALLUM W. | UNITED KINGDOM | SURGEON | X |  |
| 1. BAGNACCI, C. | ITALY | RADIOLOGIST |  | X |
| 1. BAIOCCHI, G. | ITALY | SURGEON | X | X |
| 1. BENCIVENGA, M. | ITALY | SURGEON | X | X |
| 1. BERLTH, F. | GERMANY | SURGEON |  | X |
| 1. BORGNO, L. | URUGUAY | SURGEON | X |  |
| 1. CACCIALANZA, R. | ITALY | NUTRITIONIST | X |  |
| 1. CARNEIRO, F | PORTUGAL | PATHOLOGIST |  | X |
| 1. CASELLA, F. | ITALY | SURGEON | X |  |
| 1. CASTELLI, C. | ITALY | PATHOLOGIST |  | X |
| 1. CASTRO, O | BRAZIL | SURGEON | X | X |
| 1. CORSO, S. | ITALY | MOLECULAR BIOLOGIST |  | X |
| 1. D’UGO, D. | ITALY | SURGEON |  | X |
| 1. DA COSTA, P. | PORTUGAL | SURGEON | X | X |
| 1. DAL CERO, MG. | SPAIN | SURGEON |  | X |
| 1. DE GIULI, M. | ITALY | SURGEON | X |  |
| 1. DE MANZONI, G. | ITALY | SURGEON | X | X |
| 1. DE PASCALE | ITALY | SURGEON | X | X |
| 1. DERKS, S. | NETHERLANDS | ONCOLOGIST |  | X |
| 1. DI BARTOLOMEO, M. | ITALY | ONCOLOGIST |  | X |
| 1. DONINI, A. | ITALY | SURGEON |  | X |
| 1. DONOHOE, C. | IRELAND | SURGEON | X |  |
| 1. DROLAIZ LIU | NETHERLANDS | PATHOLOGIST |  | X |
| 1. ERCOLANI | ITALY | SURGEON | X | X |
| 1. EVENO | FRANCE | SURGEON | X | X |
| 1. FRAMARINI, M. | ITALY | SURGEON |  | X |
| 1. FREJLICH, E. | POLAND | SURGEON |  | X |
| 1. FUMAGALLI, U. | ITALY | SURGEON | X | X |
| 1. GIACOPUZZI, S. | ITALY | SURGEON | X | X |
| 1. GIORDANO, S. | ITALY | MOLECULAR BIOLOGIST |  | X |
| 1. GISBERTZ, S. | NETHERLANDS | SURGEON | X | X |
| 1. GOCKEL | GERMANY | SURGEON | X | X |
| 1. GRAZIOSI, L. | ITALY | SURGEON | X | X |
| 1. HARTGRINK | GERMANY | SURGEON | X | X |
| 1. HÖLSCHER, A. | GERMANY | SURGEON |  | X |
| 1. KASSAB | BRAZIL | SURGEON | X | X |
| 1. KIELAN | POLAND | SURGEON | X | X |
| 1. KROESE T. | NETHERLANDS | SURGEON |  | X |
| 1. LANGER R. | SWITZERLAND | PATHOLOGIST |  | X |
| 1. LONARDI, S. | ITALY | ONCOLOGIST | X | X |
| 1. LORDICK | GERMANY | ONCOLOGIST | X |  |
| 1. LOURENCO | BRAZIL | SURGEON | X | X |
| 1. MALTONI, M. | ITALY | ONCOLOGIST |  | X |
| 1. MANSFIELD | USA | SURGEON | X | X |
| 1. MARANO, L. | ITALY | SURGEON |  | X |
| 1. MARINO, E. | ITALY | SURGEON |  | X |
| 1. MARKAR, S. | UNITED KINGDOM | SURGEON | X | X |
| 1. MARRELLI, D. | ITALY | SURGEON |  | X |
| 1. MARTINELLI, G. | ITALY | ONCOLOGIST | X | X |
| 1. MAZZEI M.A. | ITALY | RADIOLOGIST | X | X |
| 1. MELISI | ITALY | ONCOLOGIST | X | X |
| 1. MEYER | GERMANY | SURGEON | X | X |
| 1. MILANDRI | ITALY | ONCOLOGIST |  | X |
| 1. MINISTRINI, S. | ITALY | SURGEON | X | X |
| 1. MOLINARI, C | ITALY | MOLECULAR BIOLOGIST |  | X |
| 1. MONIG | SWITZERLAND | SURGEON |  | X |
| 1. MORGAGNI, P | ITALY | SURGEON | X | X |
| 1. MOSTERT | NETHERLANDS | ONCOLOGIST |  | X |
| 1. MOULLA | GERMANY | SURGEON | X | X |
| 1. MURA, G. | ITALY | SURGEON | X | X |
| 1. NILSSON, M. | SWEDEN | SURGEON | X | X |
| 1. PERA, M. | SPAIN | SURGEON |  | X |
| 1. PIESSEN | FRANCE | SURGEON | X |  |
| 1. POLKOWSKI | POLAND | SURGEON | X | X |
| 1. QUINZII, A. | ITALY | ONCOLOGIST |  | X |
| 1. RAPPOSELLI, I. | ITALY | ONCOLOGIST |  | X |
| 1. RAUSEI, S. | ITALY | SURGEON |  | X |
| 1. REDDAVID | ITALY | SURGEON | X |  |
| 1. RENAUD, F. | FRANCE | PATHOLOGIST |  |  |
| 1. REYNOLDS | IRELAND | SURGEON | X |  |
| 1. ROSA | ITALY | SURGEON | X | X |
| 1. ROSATI | ITALY | SURGEON | X | X |
| 1. ROVIELLO F. | ITALY | SURGEON | X |  |
| 1. ROVIELLO G. | ITALY | ONCOLOGIST | X |  |
| 1. RUDNO-RUDZINSKA, J. | POLAND | SURGEON |  | X |
| 1. SACCO, M. | ITALY | SURGEON | X |  |
| 1. SALATI, M. | ITALY | ONCOLOGIST |  | X |
| 1. SANTOS, L. | PORTUGAL | SURGEON | X | X |
| 1. SARAGONI, L. | ITALY | PATHOLOGIST |  | X |
| 1. SCHNEIDER | SWITZERLAND | SURGEON |  | X |
| 1. SO, J. | SINGAPORE | SURGEON | X | X |
| 1. SOLAINI, L. | ITALY | SURGEON |  | X |
| 1. SUNNY KIM | KOREA | SURGEON | X | X |
| 1. TERASHIMA, M. | JAPAN | SURGEON | X | X |
| 1. TIBERIO | ITALY | SURGEON | X | X |
| 1. TOMEZZOLI, A | ITALY | PATHOLOGIST |  | X |
| 1. VALGIUSTI | ITALY | SURGEON | X | X |
| 1. VAN BERGE HONEGOUWEN | NETHERLANDS | SURGEON |  | X |
| 1. VAN HILLEGESBERGE | NETHERLANDS | SURGEON | X | X |
| 1. VIETH | GERMANY | PATHOLOGIST | X |  |
| 1. WESTON A. | BRAZIL | SURGEON |  | X |
| 1. WIJNHOVEN | NETHERLANDS | SURGEON | X | X |
| 1. YANG | KOREA | SURGEON | X | X |
| TOTAL |  |  | 53 | 78 |
